# Identification of *Peroxiredoxin* (*PRX*) Genes from Pepper Fruits: Involvement in Ripening and Modulation by Nitric Oxide (NO)

**DOI:** 10.3390/antiox14070817

**Published:** 2025-07-02

**Authors:** Fátima Ramírez-Mellado, Salvador González-Gordo, José M. Palma, Francisco J. Corpas

**Affiliations:** Group of Antioxidants, Free Radicals and Nitric Oxide in Biotechnology, Food and Agriculture, Department of Stress, Development and Signaling in Plants, Estación Experimental del Zaidín, Spanish National Research Council (CSIC), C/Profesor Albareda 1, 18008 Granada, Spain; fatimarm@correo.ugr.es (F.R.-M.); salvador.gonzalez@eez.csic.es (S.G.-G.); josemanuel.palma@eez.csic.es (J.M.P.)

**Keywords:** antioxidants, pepper fruit, ripening, S-nitrosation

## Abstract

Peroxiredoxins (Prxs; EC 1.11.1.15) are a group of thiol peroxidases that catalyze the detoxification of H_2_O_2_ and other organic hydroperoxides. The ripening of pepper (*Capsicum annuum* L.) fruit involves significant phenotypic, physiological, and biochemical changes. Based on the available pepper plant genome, eight *PRX* genes were identified and named *CaPRX1*, *CaPRX1-Cys*, *CaPRX2B*, *CaPRX2E*, *CaPRX2F*, *CaPRX2-CysBAS1*, *CaPRX2-CysBAS2*, and *CaPRX Q*. Among these, only CaPRX1-Cys was not detected in the transcriptome (RNA-Seq) of sweet pepper fruits reported previously. This study analyzes the modulation of these seven *CaPRX* genes during ripening and after treating fruits with nitric oxide (NO) gas. A time-course expression analysis of sweet pepper fruit during ripening revealed that two genes were upregulated (*CaPRX1* and *CaPRX2E*), two were downregulated (*CaPRX2B* and *PRX Q*), and three were unaffected (*CaPRX2F*, *CaPRX2-CysBAS1*, and *CaPRX2-CysBAS2*). Gene expression was also studied in three hot pepper varieties with varying capsaicin contents (*Piquillo* < *Padrón* < *Alegría riojana*), showing a differential expression pattern during ripening. Furthermore, NO treatment of sweet pepper fruits triggered the upregulation of *CaPRX2B* and *CaPRXQ* genes and the downregulation of *CaPRX1* and *CaPRX2-CysBAS1* genes, while the other three remained unaffected. Among the CaPrx proteins, four (*CaPrx2B*, *CaPrx2-CysBAS1*, *CaPrx2-CysBAS2*, and *CaPrx2E*) were identified as susceptible to *S*-nitrosation, as determined by immunoprecipitation assays with an antibody against *S*-nitrocysteine and further mass spectrometry analyses. These findings indicate the diversification of *PRX* genes in pepper fruits and how some of them are regulated by NO, either at the level of gene expression or through protein *S*-nitrosation, a NO-promoting post-translational modification (PTM). Given that Prxs play a crucial role in stress tolerance, these data suggest that Prxs are vital components of the antioxidant system during pepper fruit ripening, an event that is accompanied by physiological nitro-oxidative stress.

## 1. Introduction

Peroxiredoxins (Prxs) constitute a group of thiol peroxidases that catalyze the detoxification of H_2_O_2_ and other organic hydroperoxides (ROOH), as well as peroxynitrite (ONOO^−^) [1,2,3]. Prxs are widely distributed in practically all living organisms [4,5,6]. Considering their different subunits, positions, and the number of conserved cysteine residues in their sequence, Prxs can be classified into different subclasses [7,8]. In higher plants, up to four different groups have been described: Prx 1-Cys, Prx 2-Cys, Prx II, and Prx Q. They are encoded by a family of genes made up of at least nine members, located in several subcellular compartments [9,10,11]. The metabolic functions of the different Prxs depend on their subcellular location. Prx 1-Cys contains a target signal sequence that exports the protein to the nucleus, whereas the N-terminus of Prx 2-Cys is very important for importing the protein into the chloroplast [12]. On the other hand, the sequence of the Prx II subclass lacks signal peptides and they are present in most subcellular compartments. Lastly, Prx Q shows an extension at its N-terminus that determines its chloroplastid location [13,14]. Although the functions of all subclasses have not been fully characterized, Prxs play a very important role in scavenging peroxides, thus constituting a protective barrier against oxidative stress processes [15,16]. With catalase and other peroxidases’ activities, i.e., ascorbate peroxidase (APX), Prxs participate in signal transduction processes during plant development by regulating intracellular levels of H_2_O_2_ [17]. In other cases, it has been described that the Prxs act as chaperones [18], participating in the regulation of other enzymes that act as redox sensors in the cell [3,19].

Pepper fruits, along with tomatoes, belong to the Solanaceae family and are widely consumed horticultural fruits with significant economic importance [20,21,22]. Nutritionally, peppers are rich in antioxidants, with vitamin C being the most abundant [23,24,25]. Pepper fruits are typically categorized as either sweet or hot based on their capsaicin content, which is an exclusive compound of the genus *Capsicum*. Thus, pepper varieties that do not produce capsaicin, due to the absence of certain genes involved in its biosynthesis, are referred to as sweet peppers. As pepper fruits ripen, various changes occur at the transcriptomic, proteomic, and metabolomic levels [26,27,28]. The most noticeable change at the phenotypic level is the transition from green in immature fruits to red, yellow, orange, or purple in ripe peppers [29,30,31,32]. Recent research has shown that ripening induces nitro-oxidative stress, impacting the metabolism of reactive oxygen and nitrogen species (ROS and RNS, respectively) [33,34,35,36]. Antioxidant systems such as ascorbate peroxidase, catalase, superoxide dismutase, and type III peroxidase are regulated during ripening and by nitric oxide (NO) [37,38,39]. However, information related to Prxs in pepper fruit is currently scarce. This study aims to identify and characterize the genes that code for Prxs in pepper fruits, to investigate how they are modulated during ripening in sweet and hot peppers, and to analyze their modulation by the effect of the application of exogenous NO. Globally, only four out of the eight identified CaPrxs are susceptible to *S*-nitrosation, a post-translational modification (PTM) promoted by NO.

## 2. Materials and Methods

### 2.1. Plant Material

Different varieties of pepper (*Capsicum annuum* L.) fruits have been used, including sweet and hot types. The sweet pepper fruits, California-type Melchor cultivar, were collected from experimental greenhouses of the company Syngenta Seeds Ltd. (El Ejido, Almería, Spain). They underwent a selection process to ensure similar size, color, and shape. The experimental treatment involved peppers at different stages of maturation: immature (green), breakpoint (BP), and ripe (red). The hot pepper fruits were taken as immature (green) and ripe (red) from the Padrón, Piquillo, and Alegría riojana varieties, provided by their respective regulatory entities of the Protected Designation of Origin of “Pemento de Herbón” (La Coruña, Spain) and “Piquillo de Lodosa” (Navarra, Spain).

### 2.2. Identification of PRX Genes Members

The genes encoding for Prxs in sweet pepper were identified using the National Center for Biotechnology Information (NCBI) database (https://www.ncbi.nlm.nih.gov/) (accessed on 20 February 2024), with the pepper genome accession number UCD10Xv1.1, established by the University of California in 2018 [40] as reference. Utilizing the NCBI BLASTp version 2.16.0+ tool and referencing the genes and proteins of *Arabidopsis thaliana* [7] and rice (*Oryza sativa*) [41], the genes and resulting proteins for Prxs were searched for based on homology with the pepper proteome. The amino acid sequences of the proteins were obtained from NCBI (accessed on 20 February 2024). Several criteria were set to determine the candidate sequences for PRXs in pepper (*CaPRXs*), including identity (identity) ≥ 70%, sequence coverage (query coverage) ≥ 98%, and a threshold value (threshold value) ≤ 10^−42^. Additionally, conserved domains characteristic of Prxs, such as the thioredoxin domain (IPR013766), the hydroperoxide alkyl reductase subunit (AhpC/TSA) (IPR000866) containing the catalytic cysteine for the reduction in hydroperoxides, and the presence of C-terminus (IPR019479) protecting cells from membrane oxidation, were determined. These domains were identified through the InterPro database (https://www.uniprot.org/) (accessed on 5 March 2024) [42].

The chromosomal location of the genes encoding for Prxs was obtained from NCBI. Utilizing the MG2C v2.1 program (http://mg2c.iask.in/mg2c_v2.1/) (accessed on 5 March 2024) [43], the chromosomal map of these genes within the sweet pepper genome could be created.

### 2.3. Analysis of Gene Structure and Regulatory Elements

To analyze the structure of each of the gene’s coding for Prxs, information on their composition was extracted regarding the number of exons and introns, as well as the 5′ and 3′ UTR regulatory regions from the NCBI Nucleotide database (https://www.ncbi.nlm.nih.gov/nucleotide/ (accessed on 6 March 2024).

On the other hand, the regulatory elements that may intervene in the expression of these genes were also analyzed. To do this, the 2000 base pairs (bp) upstream of the transcription start point were obtained, since those elements that regulate gene expression are usually located in this region. This information was obtained from NCBI Nucleotide (https://www.ncbi.nlm.nih.gov/nucleotide/) (accessed on 12 March 2024). From these sequences, using the PlantCARE tool (https://bioinformatics.psb.ugent.be/webtools/plantcare/html/) (accessed on 12 March 2024), it was possible to obtain the regulatory elements (cis-acting regulatory elements) present in these sequences, which were visualized using the TBTools v2.069 program within the “HeatMap” function [44].

### 2.4. Analysis of Localization and Protein Characteristics

Using the WoLF PSORT online tool (https://wolfpsort.hgc.jp/) (accessed on 14 March 2024) [45], the prediction of the subcellular localization of the Prxs was obtained thanks to the analysis of their amino acid sequence.

Among the Prx protein characteristics analyzed, molecular weight and isoelectric point were taken into account. The predictions of these characteristics were obtained through the amino acid sequence of each Prx protein using the Compute pI/MW tool of the Expasy online server (https://www.expasy.org/) (accessed on 14 March 2024) [46].

### 2.5. Phylogenetic Analysis and Conserved Motifs

From the amino acid sequence of the Prxs and through the online tool MEME-SUITE version 5.5.7 [47], the conserved motifs of these proteins were located. They were subsequently visualized with the TBTools v2.069 program [44].

The analysis and construction of the phylogenetic tree of the Prxs was carried out by searching for orthologous proteins in different plant species, in turn carrying out sequence alignments using the Clustal Omega version 1.2.4 online tool of the EMBL-EBI (European Bioinformatics Institute of the European Molecular Biology Laboratory) (https://www.ebi.ac.uk/jdispatcher/msa/clustalo) (accessed on 14 March 2024). The sequences used are collected in the Supplementary Materials. Using the MEGA11 program [48], the phylogenetic tree of the collected sequences was built with a criterion of maximum similarity, maintaining the default parameters provided by the program.

### 2.6. In Silico Analysis of Possible Protein–Protein Interactions

By using the STRING database v.12., and through the protein sequence, the possible protein–protein interactions between all the Prxs present in the sweet pepper proteome were obtained (https://string-db.org/, accessed on 6 March 2024) [49].

### 2.7. Treatment of Sweet Pepper Fruits in an Environment Enriched in Nitric Oxide (NO)

The sweet pepper fruits of the California Melchor type were exposed to an atmosphere enriched with NO, following the method described in [38,50], as they are the most commercially important variety in southeastern Spain. From the same pepper plant, fruits were taken at three stages: immature (green), breaking point (BP1), and ripe (red). Additionally, the BP1 group underwent two different treatments, resulting in a control group for break point fruits not treated with NO (BP2 − NO) and a treated group with NO at 5 ppm for 60 min (BP2 + NO). The NO-enriched environment was created using the method outlined in [50] (see Supplementary Figure S1). After the 60 min treatment, the fruits were left at room temperature (RT) for 3 days. Following this, fruits were processed by cutting them into small pieces, which were then frozen with liquid nitrogen and stored at −80 °C for subsequent analysis. The treatment design can be found in Supplementary Figure S2.

Differential gene expression analysis was performed using DEgenes-Hunter, a flexible R-based pipeline designed to automate RNA-seq workflows—particularly for non-model organisms [51]. This tool compares gene expression levels across samples and treatments by integrating multiple statistical algorithms, including EdgeR v4.6.2, DESeq2 version 1.48.1, Limma version 3.64.1, and NOISeq version 2.52.0, each applying distinct normalization methods and statistical frameworks to ensure robust and reliable results. Furthermore, a time-course analysis of *CaPRX* gene expression was conducted, using the expression levels in green fruits (G) as the reference point.

### 2.8. Protein Extraction from Pepper Fruits

The plant material stored at −80 °C was ground to a powder texture using an IKA^®^ A11Basic analytical mill (IKA^®^, Staufen, Germany), and a 1:1 ratio of fruit material and buffer [100 mM Tris-HCl buffer, pH 8.0, containing 0.1% (*v*/*v*) Triton X-100, 1 mM ethylenediaminetetraacetic acid (EDTA), 10% (*v*/*v*) glycerol] was used for protein extraction. The buffer mixture with the plant material was kept at 4 °C and constantly shaken until completely thawed. After this, the plant extract obtained was transferred to 1.5 mL tubes and centrifuged at 4 °C for 20 min at 20,000× *g*. Then, the supernatant obtained after centrifugation was collected and used for further analyses. Protein concentration was determined using the Bio-Rad protein assay (Hercules, CA, USA), with bovine serum albumin as standard.

### 2.9. Immunoprecipitation of Pepper S-Nitrosated Protein with Magnetic Particles

The process of immunoprecipitating proteins with magnetic particles was performed using the PureProteome Protein A and Protein G Magnetic Beads (Millipore, Darmstadt, Germany) following the instructions provided by the manufacturer. Briefly, the solution containing Protein A-bound magnetic beads (100 μL) was transferred to a 1.5 mL tube. The beads were trapped with a magnet rack and the suspended solution was then removed. After that, the beads underwent 3 washes with PBS-T (PBS + 0.1% Tween 20). Following the washes, the beads were incubated in constant motion with 5 μg of commercial *S*-nitrosocysteine antibody in a total volume of 200 μL completed with PBS for 10 min at room temperature. After the incubation, the magnetized beads were separated, and the supernatant was removed. Subsequently, the beads underwent 3 washes with PBS-T, and 500 μL of pepper fruit protein extract was added. The extract binds specifically to the antibody bound to the magnetic particles. The extract together with the beads was incubated for 1 h at room temperature with constant shaking. After incubation, the supernatant was removed, and the beads were washed 3 times with TBS-T. Following the removal of the supernatant, thus leaving only the magnetic beads to which our proteins were attached, 40 μL of commercial 1x Laemmli buffer (BioRad, Basel, Switzerland). The mixture was then incubated at 70 °C for 10 min. After this, the beads were magnetized again, and the supernatant, containing the immunoprecipitated proteins, was collected. Finally, the sample was either used immediately or stored at 4 °C for further use.

### 2.10. Electrophoresis in Polyacrylamide Gels Under Denaturing Conditions and Immunoblotting Analysis

Proteins were separated by SDS-PAGE in gradient (4–20%) polyacrylamide gels according to the manufacturer (BioRad, Mini-PROTEAN TGX). The commercial Precision Plus ProteinTM Dual Color Standards (BioRad) was used as a marker. After electrophoresis, the polyacrylamide gels were stained with commercial Coomassie blue (BioRad) solutions. Alternatively, after electrophoresis, the gel was transferred to a commercial Trans-Blot Turbo PVDF membrane with a pore size of 0.2 μm (BioRad). Blotting was carried out using the Trans-Blot Turbo apparatus at a 25 V setting for 7 min. The transferred membrane was then blocked with 50 mL of blocking solution (0.5% skimmed cow’s milk powder in TBS-T). After blocking, the membrane was washed with TBS-T (TBS + 1% Tween 20) for 10 min, three times. Next, the membrane was incubated overnight with the primary antibody against yeast (*Saccharomyces cerevisiae*) Prx1p [52,53] at an optimal dilution for each sample, with continuous shaking and at 4 °C. After incubation, the membrane was washed with TBS-T for 10 min, three times. The membrane was then incubated with the horseradish peroxidase-conjugated secondary antibody, which was specifically bound to the primary antibody at a specific dilution for each condition, for 1 h at room temperature with shaking. Subsequently, the membrane was subjected to three washes of 10 min each with TBS-T before being visualized. For visualization, the commercial Clarity Western ECL substrate development solution (BioRad) was used, and chemiluminescence was detected with the “ChemiDoc XRS+ Gel Imaging System” (BioRad).

### 2.11. Mass Spectrometry Analysis of S-Nitrosated Protein Obtained by Immunoprecipitation

For mass spectrometry analysis, immunoprecipitated samples with an antibody against *S*-nitrosocysteine were suspended in 40 µL of 1× Laemmli buffer containing 32.4 mM Tris-HCl (pH 6.8), 1% (*w*/*v*) SDS, and 13.5% (*w*/*v*) glycerol. The samples were then digested on a solid support using PROTIFI STRAP columns, which resulted in clean tryptic peptides, ready for analysis. Subsequently, the peptides were analyzed by liquid (LC-MS) using a short gradient, as previously described [54].

## 3. Results

### 3.1. Identification of Genes Encoding Prxs and Their Chromosomal Localization

Prxs are thiol-dependent peroxidase proteins [55] with redox activity, capable of acting against various substrates [56]. Referring to the information on Prxs from *A. thaliana* plants [56] and rice [41], the Prxs present in this study were identified through homology in the pepper genome. Specifically, eight genes encoding these proteins were identified and named *CaPRX2B*, *CaPRX2F*, *CaPRX2*-*Cys BAS1*, *CaPRX1*, Ca*PRX*Q, *CaPRX1-Cys1*, Ca*PRX*2E, and *CaPrx2-Cys BAS2*. Table 1 shows the main characteristics of the genes and their encoded proteins. Data obtained from the transcriptome of California-type sweet peppers [57] indicate that all these genes, except *CaPRX1-Cys*, are expressed in sweet pepper fruits.

### 3.2. Analysis of CaPRX Gene Structure and Regulatory Elements

**Figure 1:**
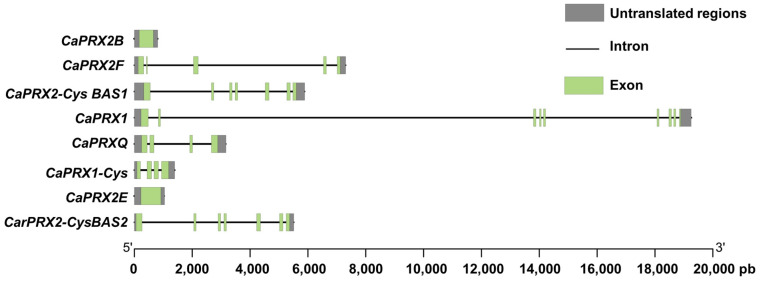
Genomic organization of the pepper *CaPRX* gene family. The gene structure is illustrated with exons represented by green boxes, introns by black lines, and untranslated regions by gray boxes. Exon–intron regions are drawn to scale.

The structure of the genes encoding CaPrxs is illustrated in Figure 1. As it is shown, each gene has a distinct structure. Only two genes, *CaPRX2B* and *CaPRX2E*, consist of a single exon, each less than 2000 base pairs (bp) long. The remaining genes have multiple exons separated by introns. Specifically, both *CaPRXQ* and *CaPRX1-Cys1* have four exons and three introns; *CaPRX2F* has five exons and four introns; *CaPRX2-Cys BAS1* and *CaPrx2-Cys BAS2* consist of seven exons and six introns. The longest gene, *CaPRX1*, is noteworthy for having nine exons and eight introns, with a length of about 20,000 bp. All of the Prx-encoding genes comprise 5′UTR and 3′UTR regions, which are untranslated.

**Figure 2:**
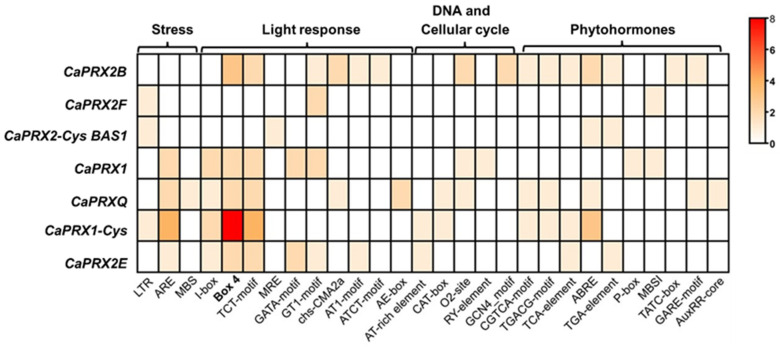
Heatmap of cis-regulatory elements in the 2000 bp region upstream of the transcription start site of *CaPRX* genes. The cis-regulatory elements are categorized based on their functional roles, including DNA regulation and cell cycle, light response, stress response, and phytohormones. The motifs were identified using the PlantCARE database.

The regulatory elements responsible for regulating gene expression have been analyzed. We set the regulatory elements present in the 2000 base pairs upstream of the start of transcription of each gene (Figure 2). Among all the elements studied, Box 4, particularly in the *CaPRX1* gene, stands out. This element is related to the fruit’s response to light. The *CaPRX-Cys BAS2* gene was not found because, when applying filters to find the most prominent elements, none were detected in the 2000 base pairs upstream of the start of transcription.

### 3.3. Analysis of Preserved Prx Protein Motifs

**Figure 3:**
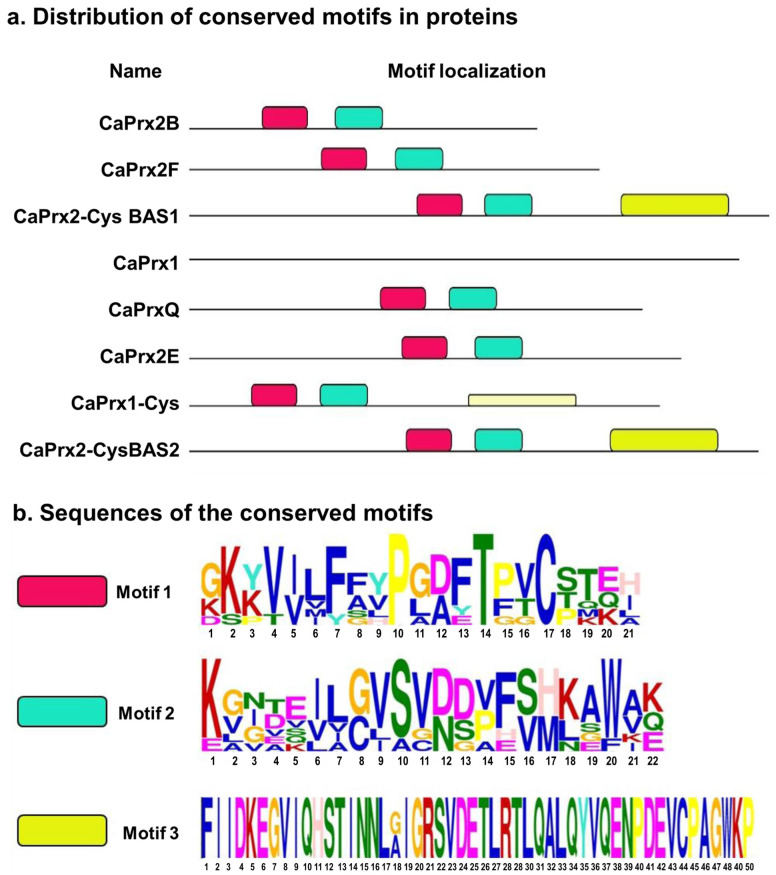
Analysis of conserved motifs in CaPrx proteins. (**a**) Representation of the distribution of conserved motifs in the proteins. (**b**) Sequences of the conserved motifs, where the higher size of each amino acid letter indicates the higher degree of conservation within the motif.

By analyzing the structure of the Prxs encoded proteins, it was possible to identify their conserved motifs (Figure 3). A total of three conserved motifs were identified in the Prxs, of which two were found in the eight Prxs identified, while the third motif was only detected in the BAS-type Prxs and, with less certainty, in the CaPrx1-Cys (Figure 3a). The amino acid sequence of the identified conserved motifs is shown in Figure 3b.

### 3.4. Phylogenetic Tree

**Figure 4:**
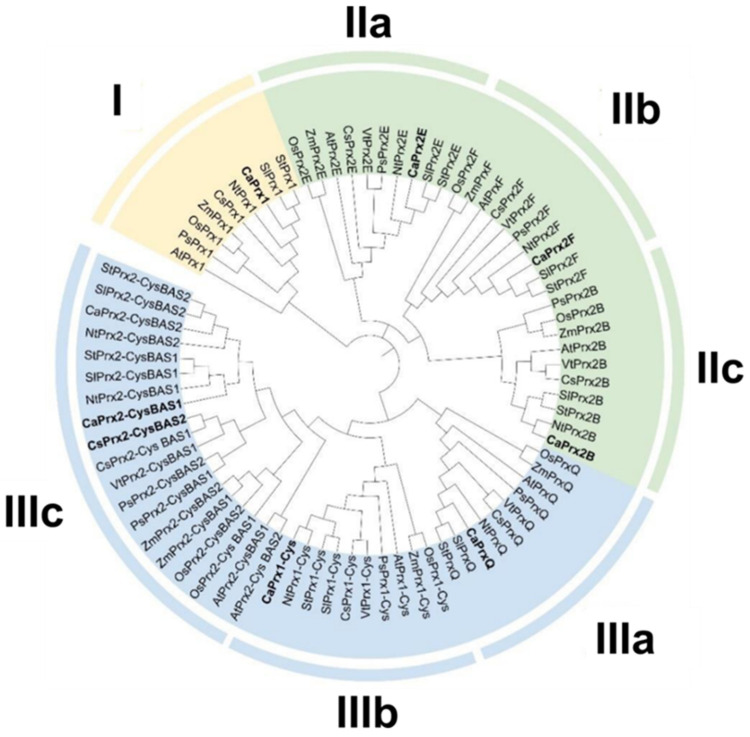
Phylogenetic tree of CaPrxs with other Prxs from different species (identifiers are listed in Supplementary Table S1). Clusters (I–III) are displayed using different colors. The CaPrxs are highlighted in bold compared to those from the other species. *Arabidopsis thaliana* (At), pepper (*Capsicum annuum*, Ca), rice (*Oryza sativa*, Os), grapevine (*Vitis vinifera*, Vt), tomato (*Solanum lycopersicum*, Sl), corn (*Zea mays*, Zm), potato (*Solanum tuberosum*, St), tobacco (*Nicotiana tabacum*, Nt), Valencian orange (*Citrus sinensis*, Cs), and poppy (*Papever somniferum*, Ps).

The phylogenetic tree in Figure 4 illustrates the relationships among CaPrx proteins and Prxs from other plant species. It highlights the degree of similarity between the Prxs and divides the tree into three main clusters (I, II, III), each containing distinct subgroups denoted by letters. Notably, the BAS-type Prxs do not form two separate groups as initially expected; instead, the phylogenetic analysis reveals a different grouping. Additionally, it is worth mentioning that the CaPrx proteins from sweet pepper are most closely related to those of tomato (*Solanum lycopersicum*) and potato (*Solanum tuberosum*), reflecting their shared lineage within the Solanaceae family.

### 3.5. Possible Protein–Protein Interactions

**Figure 5:**
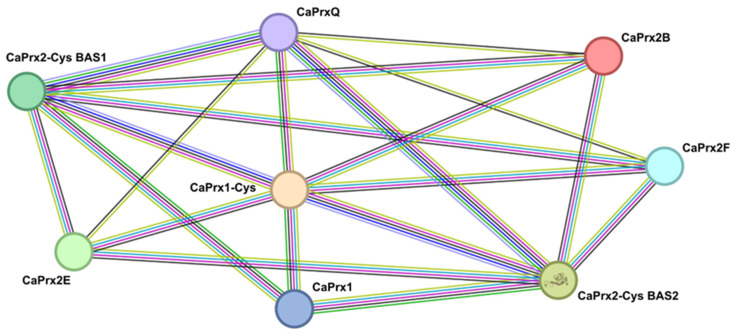
Scheme of computationally predicted interactions of CaPRXs proteins. Each type of interaction is shown in a different color: pink, experimentally determined known interaction; blue, known interaction of curated databases; green, interaction predicted by neighboring genes; dark blue, interaction predicted by gene co-occurrence; yellow, interaction extracted from the derivation of correlations between the information in the databases; black, interaction by co-expression of genes; and light blue, protein homology interaction.

Figure 5 shows the results of the computational analysis of potential protein–protein interactions among the *CaPrx* proteins identified in pepper. Notably, these proteins exhibit numerous interactions, with *CaPrx1-Cys* standing out as the most significant, as it interacts with all seven other *CaPrx* proteins.

### 3.6. Expression of CaPRXs Genes in Fruits at Ripening and After Treatment with NO

**Figure 6:**
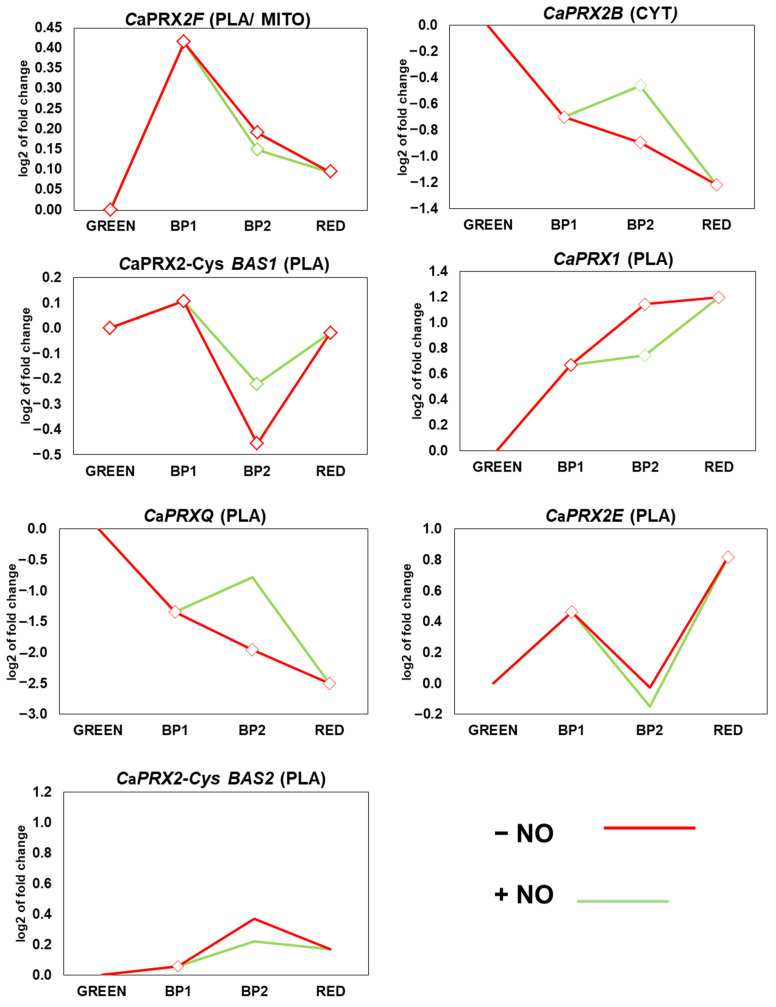
Time course expression analysis of seven *CaPRX* genes (RNA-Seq) under natural ripening conditions and after exogenous NO treatment. Samples of sweet pepper fruits at different ripening stages correspond to immature green, breaking point 1 (BP1), breaking point 2 with (green line), without (red line) NO treatment (BP2 + NO and BP − NO, respectively), and ripe red. Statistically significant changes in expression levels (*p* < 0.05) compared to green fruit are indicated with diamonds. CYT, cytosol; MIT, mitochondrim; PLA, plastid.

The analysis of the expression of the *CaPRXs* genes during the ripening process of sweet pepper fruit in an atmosphere enriched in NO is depicted in Figure 6. The genes’ expression exhibits different behaviors throughout the ripening process, except for *CaPRX1* which did not show any changes in its expression. The results indicate that the expression of the *CaPRXs* genes does not follow a common pattern. During the ripening process, *CaPRX2B* and *CaPRXQ* transcript levels declined, whereas *CaPRX1* and *CaPRX2E* were significantly upregulated. The expression of the remaining *CaPRX* genes was not significantly affected. In contrast, following NO treatment, *CaPRX2B*, *CaPRX2-CysBAS1*, and *CaPRXQ* were upregulated, while *CaPRX1* was downregulated.

### 3.7. Comparison of the Expression of CaPRX Genes in Sweet and Hot Pepper Fruits

**Figure 7:**
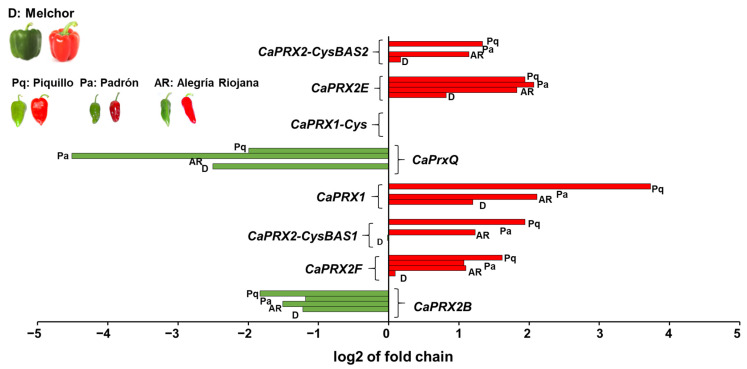
Comparison of *PRX* genes expression in both immature (green) and mature (red) fruits from several *C. annuum* varieties with different capsaicin content, including sweet pepper (without capsaicin; Melchor) and three Spanish autochthonous pepper varieties with different pungency degrees, Piquillo (Pq), Padrón (Pa), and Alegría riojana (AR).

To gain deeper insights into the physiological function of these genes, their expression was compared in fruits from sweet and hot varieties. The study analyzed the expression of *CaPRX* genes in sweet pepper fruits (California variety) and was compared with that of the same genes in other pepper hot varieties using previously obtained transcriptomes. The comparison included three varieties of hot pepper fruits autochthonous from Spain, specifically the Piquillo, Padrón, and Alegría riojana, along with the California variety (Figure 7). This analysis provided insights into the expression changes of the *CaPRX* genes throughout the ripening process in both sweet and hot pepper fruits.

The immature stage (green) was plotted by a log2 fold change with negative values, while the ripe stage (red) was displayed by a log2 fold change with positive values. The analysis revealed that most *CaPRX* genes showed higher expression in the ripe state in both sweet and hot peppers, although not all varieties exhibited the same level of increased expression. However, the *CaPRXQ* and *CaPRX2B* genes followed a different pattern, showing higher expression in the immature green state. Additionally, the *CaPRX1* gene did not show significant changes in expression during the ripening process. Again, the *CaPRX1-Cys* gene was not found in these hot pepper varieties either.

### 3.8. Immunodetection of Prxs in Pepper Fruits

**Figure 8:**
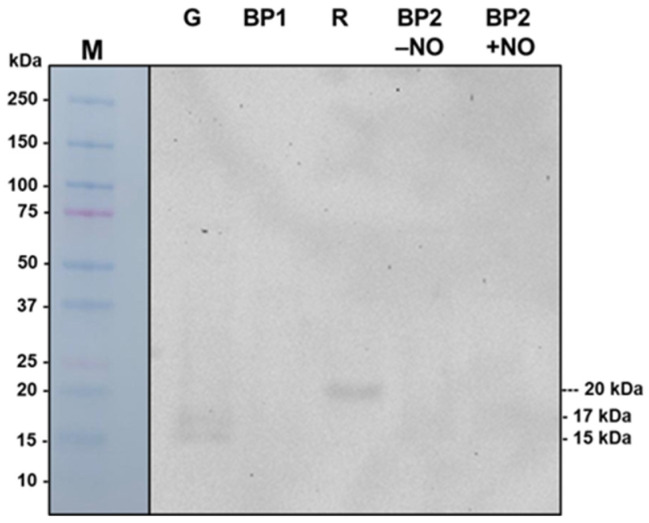
Immunodetection of CaPrxs with an antibody against yeast Prx1p (dilution 1:5000) in samples of sweet pepper fruits at different ripening stages: immature green (G), breaking point 1 (BP1), breaking point 2 with and without NO treatment (BP2 + NO and BP − NO, respectively), and ripe red (R). All lanes were loaded with 18 μg of protein.

Figure 8 shows the immunodetection of CaPrxs with an antibody against yeast Prx1p in samples of sweet pepper fruits at different ripening stages: immature green (G), breaking point 1 (BP1), breaking point 2 with and without NO treatment (BP2 + NO and BP − NO, respectively), and ripe red (R). In green pepper two bands of 15 kDa and 17 kDa were immunodetected, whereas in red pepper one single band of around 20 kDa was observed. In the rest of the samples, any band could be detected.

### 3.9. Immunoprecipitation with Magnetic Beads of S-Nitrosated Proteins and Detection of S-Nitrosated CaPrxs

**Figure 9:**
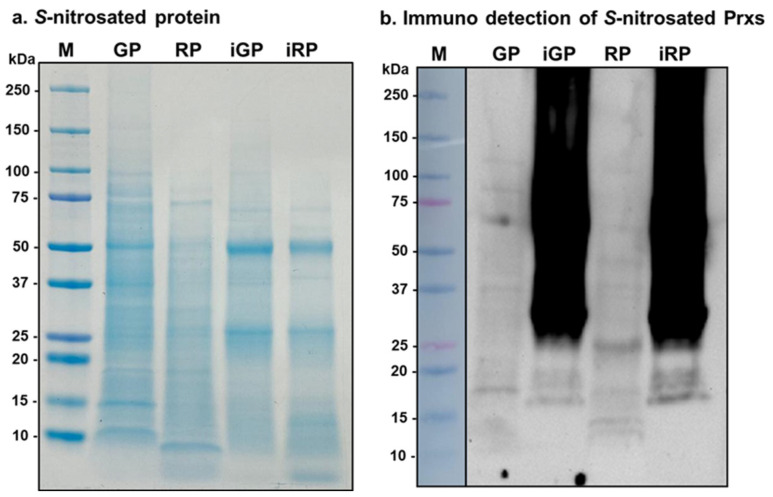
(**a**) Detection of S-nitrosated proteins from pepper fruits in acrylamide gel stained with Coomassie blue. (**b**) Immuno detection of CaPrxs among the S-nitrosated proteins using an antibody against yeast Prx1p (dilution 1:5000). Lane M: Precision Plus ProteinTM Dual Color Standards (BioRad) as a marker; Lane GP: green pepper protein extract; Lane RP: red pepper protein extract; Lanes iGP and iRP: proteins immunoprecipitated with antibody against *S*-nitrosocysteine from green and red pepper fruits, respectively. In total, 20 μg of proteins were loaded per lane.

The detection of S-nitrosated proteins in pepper fruits was carried out using the immunoprecipitation protocol with magnetic beads. To this end, after immunoprecipitation, SDS-PAGE was carried out under denaturing conditions. Figure 9a shows the whole protein profile of green and red samples, in comparison, with the immunoprecipitated *S*-nitrosated proteins (iGP and iRP). As expected, the profile of the proteins isolated using immunoprecipitation is less prominent, except for two proteins of approximately 50 and 25 kDa, which were enriched after immunoprecipitation. These likely correspond to the heavy and light chains, respectively, of the IgGs under reducing conditions which were not removed during the washing of immunoprecipitated proteins. In Figure 9b, the immunoblot probe with an antibody against Prx of the *S*-nitrosated proteins from green and red pepper fruit and the corresponding immunoprecipitates tested with an antibody against yeast Prx1p. Various immunoreactive proteins between 15 and 25 kDa (in the range of Prxs) were observed. Additionally, two very dark bands were visible in the lanes of the immunoprecipitated proteins, which corresponded to the heavy and light chains of the IgG. This effect occurred because the immunoblot was overexposed during the development process to ease the detection of the Prxs.

## 4. Discussion

Peroxiredoxins (Prxs) are thiol-dependent peroxidase proteins [55,58] capable of acting on a wide range of substrates, including hydrogen peroxide (H_2_O_2_) hydroperoxides (ROOH), and peroxynitrite (ONOOH) [59,60,61,62]. This peroxidase activity makes Prxs critical components of cellular antioxidant systems. In addition to their antioxidant role, Prxs modulate signaling pathways by participating in reactions that alter the redox state of the cell. They also function as cellular redox sensors across all organisms [7,56,63].

In plants, *Arabidopsis thaliana* was the first species where ten *PRX* genes were identified [56]. Advances in gene and protein identification, driven by ‘omics’ approaches, have since enabled the identification of *PRX* genes in other species, including *Medicago truncatula* [64], maize [65], rice [41], poplar [66], grapevine [67,68], cotton [69], and bean [70]. However, to date, no *PRX* genes have been identified in pepper fruits. This study identified a total of seven *PRX* genes in the sweet pepper transcriptome using bioinformatics tools [57]. These genes have been designated as *CaPRX2B*, *CaPRX2F*, *CaPRX2-Cys BAS1*, *CaPRX1*, *CaPRXQ*, *CaPRX1-Cys*, *CaPRX2E*, and *CaPRX2-Cys BAS2*.

The number of genes encoding Prxs in pepper matches the number found in other plant species, such as rice, which has seven *PRX*-encoding genes [67]. In addition to identifying these genes in the genome, it has been confirmed that all *CaPRX* genes are expressed in pepper fruit during the ripening process except *CaPRX1-Cys*. However, the identified genes exhibit varying expression patterns during fruit ripening as well as when they were incubated in an atmosphere enriched with NO. The opposite expression trends observed among the *CaPRX* genes could be attributed to multiple factors, including differences in gene structure (e.g., intron–exon organization), promoter elements, or regulatory motifs responsive to developmental or stress signals. Additionally, the distinct subcellular localizations and specific catalytic mechanisms of each PRX protein may reflect their specialized roles during fruit ripening. For example, 1-Cys PRXs are often linked to seed and stress responses, while 2-Cys PRXs are typically chloroplastic and associated with photosynthetic redox regulation.

The transcriptomes of other Spanish native pepper varieties (Piquillo, Alegría riojana, and Padrón) had been previously obtained. Consequently, the presence of the *CaPRX* genes in these varieties was also confirmed, and their expression was compared across the four varieties examined in this study. The analysis revealed that each gene exhibited a similar expression pattern among the different varieties. For example, genes such as *CaPRXQ* and *CaPRX2B*, which are more highly expressed in the immature green stage of the fruit, were detected in all varieties, though at varying expression levels. Similarly, genes with increased expression in the ripe stage, such as *CaPRX2*-*Cys BAS2*, *CaPRX2E*, *CaPRX1*, *CaPRX2-Cys BAS1*, and *CaPRX2F*, also showed elevated expression in that stage across all varieties. These results suggest that *PRX* genes play fundamental roles in the ripening process of pepper fruits because they function as key components of the antioxidant defense system that helps maintain redox balance during this process. Pepper fruit ripening is known to involve elevated levels of reactive oxygen and nitrogen species (ROS and RNS), leading to a state of nitro-oxidative stress [71]. In this context, PRXs help to regulate H_2_O_2_ content, thereby protecting cellular structures and signaling networks. The regulation of *PRX* genes by NO, as observed in the upregulation of *CaPRX2B* and *CaPRXQ*, and downregulation of *CaPRX1* and *CaPRX2-CysBAS1*, highlights their dynamic role during ripening. Moreover, the finding that several PRX proteins are susceptible to *S*-nitrosation, a redox-related post-translational modification mediated by NO, further suggests that NO not only affects their expression but may also modulate their activity and function at the protein level. Altogether, these results indicate that PRXs are not only responsive to NO signaling but may also act as redox sensors and regulators. This dual level of regulation—transcriptional and post-translational, supports the idea that PRXs contribute to maintaining redox homeostasis and stress adaptation during the complex physiological process of fruit ripening.

After the identification of the *CaPRXs* genes, their structure was analyzed to determine their length and organization. The results revealed that the genes exhibit a diverse range of structures. Among them, only two (*CaPRX2B* and *CaPRX2E*) have a single intron containing the coding protein information. The others consist of a succession of multiple introns and exons, with some genes having up to nine exons and eight introns. Thus, genes with more introns are generally considered to have greater potential for alternative splicing, enabling the production of multiple protein isoforms from a single gene. Additionally, the presence of more introns may contribute to increased regulatory complexity, potentially allowing for tissue-specific or stress-responsive gene expression. Analysis of the regulatory elements within the 2000 bp upstream of the transcription start site showed the presence of Box 4 in five of the eight identified genes. Notably, the *PRX1-Cys* gene contains eight copies of this transcription element. Box 4 is a transcription factor associated with responses to light [72,73], what suggest the pepper phenology associated with seasonal cycles.

The analysis of protein–protein interactions of CaPrxs using bioinformatics methods revealed a significant relationship among all the CaPrxs in pepper. Among these, CaPrx1-Cys emerges as a central hub, exhibiting predicted interactions with all seven other CaPrx proteins. This extensive connectivity suggests that CaPrx1-Cys may play a pivotal coordinating role within the peroxiredoxin network, potentially acting as a regulatory node that integrates redox signals during key physiological processes, such as fruit development and stress responses. Its central position implies that alterations in CaPrx1-Cys expression or activity could have cascading effects on the overall redox balance and antioxidant capacity in pepper tissues.

The phylogenetic tree was constructed using Prx protein sequences from different plant species, which can be found in the Supplementary Table S1. The tree revealed seven distinct groups representing each type of Prx found in pepper: Prx1, Prx1-Cys, Prx2B, Prx2E, Prx2F, PrxQ, and Prx BAS. Interestingly, in the tree, the two types of Prx BAS (BAS1 and BAS2) did not appear as separate groups, indicating a high degree of similarity between these two proteins. This might be due to their shared subgroup within Prxs. However, a consistent pattern was observed in the species closest to pepper, such as potato and tomato, which was expected as all three species belong to the same family, the Solanaceae [74].

The conserved motifs identified using bioinformatics methods align with the expected motifs in Prxs. These include the thioredoxin domain motif and the hydroperoxide alkyl reductase subunit (AhpC/TSA), which is responsible for carrying the protein’s catalytic cysteine [75]. The presence of the C-terminal has only been found in BAS-type Prxs, which protects cells from membrane oxidation and carries an extra cysteine for the molecule’s dimerization [76,77].

The results from immunoblots of pepper fruits using an antibody against yeast Prx1p [53] revealed the presence of two immunorelated proteins in pepper fruits. One was around 18 kDa, possibly corresponding to CaPrx2B, and the other was around 20 kDa, possibly corresponding to CaPrx2F. However, these results cannot be directly extrapolated, and more in-depth studies are necessary for their identification.

The selection of *S*-nitrosated proteins obtained by immunoprecipitation with magnetic particles and their identification by mass spectrometry allowed identifying CaPrx2E. This data is in good agreement with a previous report where the Prx2E was also identified by matrix-assisted laser desorption ionization-time of flight (MALDI-TOF/TOF) among a group of *S*-nitrosated proteins using another experimental approach using diaminofluorescein gels [78].

The alignment of the protein-coding for Prx2E in pepper with Arabidopsis peroxiredoxin II E (Q949U7) which was demonstrated to be *S*-nitrosated at Cys121, showed that this Cys is within a fragment of 16 amino acids. This sequence, L_110_FAVPGAFTPTCSQKH_125_, exhibits 100% identity between both proteins, with this Cys121 corresponding to the catalytic cysteine, and its *S*-nitrosation triggered the activity inhibition [79]. Furthermore, this sequence is completely conserved in other PRX 2E-2 proteins from different species, including tomato (*Solanum tuberosum*; XP_006341173.1), pomegranate (*Punica granatum*; XP_031396643.1), and apple (*Malus domestica*; XP_050122661.1), among others.

As previously mentioned, Arabidopsis Prx2E is inhibited by *S*-nitrosation. This peroxiredoxin also detoxifies peroxynitrite (ONOO^−^) and participates in trans-nitrosation processes [79,80]. Peroxynitrite is a highly reactive molecule implicated in protein nitration [81,82,83,84]. Based on these findings, it can be hypothesized that CaPrx2E, when *S*-nitrosated, becomes inhibited, leading to increased ONOO^−^ levels and, consequently, enhanced protein nitration. Indeed, prior studies have shown that pepper fruit ripening is associated with nitro-oxidative stress, including a marked rise in protein nitration [57,85]. Catalase, notably nitrated at Tyr348 and Tyr360, is among the main targets, and this modification results in its inhibition, contributing to elevated H_2_O_2_ levels [37,85]. Thus, nitric oxide (NO) appears to act upstream in H_2_O_2_ metabolism by modulating antioxidant enzymes such as Prx and catalase. 

**Figure 10:**
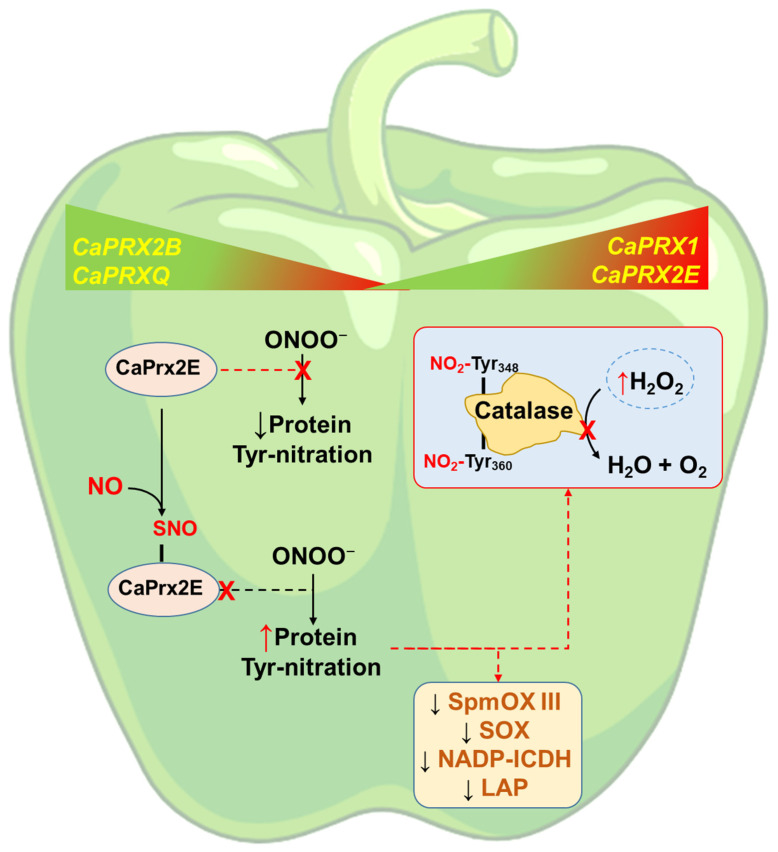
Working model showing that *CaPRX* genes are either upregulated or downregulated during the ripening of pepper fruit. It is also indicated that the *S*-nitrosation of CaPrx2E leads to its inhibition, which may result in an increase in peroxynitrite (ONOO^−^) and, consequently, in protein tyrosine (Tyr) nitration [85]. Among the proteins that undergo nitration at Tyr348 and Tyr360 is catalase [37], whose inhibition subsequently leads to an increase in H_2_O_2_ levels. Other target enzymes of nitration include NADP-isocitrate dehydrogenase (NADP-ICDH), leucine aminopeptidase (LAP) spermidine polyamine oxidase (SpmOX) III, or sulfite oxidase (SOX), all of which exhibit decreased activity due to this modification, thereby affecting multiple metabolic pathways. The red X indicates that the process is blocked.

H_2_O_2_levels play a critical role in fruit ripening, influencing whether the process is delayed or accelerated, depending on the fruit type [86,87,88,89,90,91]. Figure 10 illustrates a working model summarizing the principal findings of this study and highlights how NO, through posttranslational modifications like S-nitrosation and nitration, regulates reactive oxygen species (ROS) metabolism. This regulation affects specific proteins such as CaPrx2E and catalase, thereby impacting H_2_O_2_content and fruit ripening. Moreover, peroxynitrite levels influence the extent of nitration in various enzymes involved in redox metabolism, including NADP-isocitrate dehydrogenase (which provides NADPH), leucine aminopeptidase (LAP), spermine polyamine oxidase III (which produces H_2_O_2_during polyamine catabolism), and sulfite oxidase (which catalyzes the conversion of sulfite to sulfate) [54,92,93,94].

## 5. Conclusions

Taken together, the data obtained have enabled the identification, for the first time, of genes encoding peroxiredoxins in pepper fruits and how they are differentially regulated during ripening and in response to the exogenous application of NO. Furthermore, combined immunological and LC/MS analyses identified CaPrx2E as a target of *S*-nitrosation which suggests that NO may function upstream of ROS metabolism. Altogether, these findings reflect the complex interplay between ROS and RNS metabolism during pepper fruit ripening, in which peroxiredoxins (Prxs) play a central and essential role.

## Figures and Tables

**Table 1:** Summary of the eight *peroxiredoxin* (*PRX*) genes identified in the pepper (*C. annuum* L.) genome and some of the properties related to the protein encoded by these genes, including the number of amino acids (aa), molecular mass (kDa), theoretical pI, and their putative subcellular localization.

Gene Name	Gene ID	Chr	Protein ID	Length (aa)	Mw (kDa)	pI	Subcellular Localization
*PRX2B*	107840788	1	XP_016540190.1	162	17.41	5.80	Cytosol
*PRX2F*	107841069	1	XP_016540565.1	191	20.78	9.00	Plastid
*PRX2-CysBAS1*	107846522	1	XP_016546376.1	270	29.61	7.57	Plastid
*PRX1*	107867717	4	XP_016569570.1	256	28.47	9.53	Plastid
*PRX Q*	107877928	7	XP_016580227.2	211	23.18	9.60	Plastid
*PRX1-Cys*	107848474	9	XP_016548730.1	219	24.31	6.45	Cytosol/Nucleus
*PRX2E*	107843634	10	XP_016543463.1	229	24.60	8.26	Plastid
*PRX2-CysBAS2*	107843729	10	XP_016543590.1	265	29.04	6.75	Plastid

## Data Availability

Sequence Read Archive (SRA) data are available at the following link https://www.ncbi.nlm.nih.gov/sra/PRJNA668052 (accessed on 28 May 2020).

## Supplementary Materials

The following supporting information can be downloaded at: https://www.mdpi.com/article/10.3390/antiox14070817/s1, References [7,9,39,41,50,95,96] are cited in Supplementary Materials. Figure S1: System diagram for NO treatment of pepper fruits; Figure S2: Experimental design used in this study with the representative phenotype of sweet pepper fruits at different stages and treatments. Table S1: Identifiers of the different Prxs of different species used to develop the phylogenetic tree of the Prxs proteins.
