# Supplementary material for: Identification of Peroxiredoxin (PRX) Genes from Pepper Fruits: Involvement in Ripening and Modulation by Nitric Oxide (NO)
Source: Antioxidants (Basel). 2025 Jul 2;14(7):817. doi: 10.3390/antiox14070817 (PMC12291961; doi:10.3390/antiox14070817)
Supplement: Supplementary file 1 [file antioxidants-14-00817-s001.zip › antioxidants-3686071-supplementary.pdf]

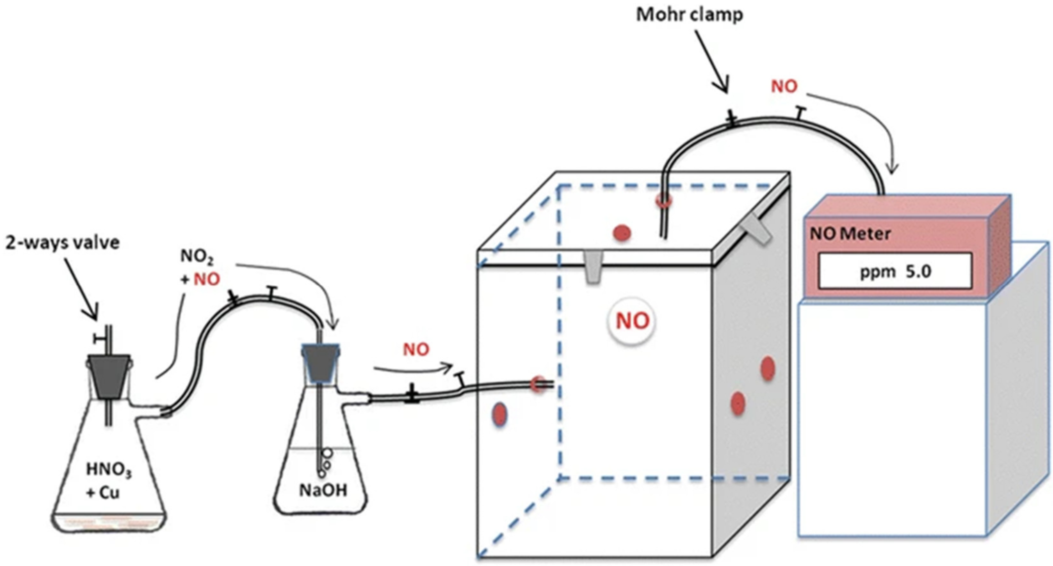

Figure S1. A comprehensive diagram of the entire system is shown, featuring pepper fruits at their breaking point placed inside an incubation chamber where they are exposed to nitric oxide (NO) gas. The NO gas is generated through a reaction between nitric acid (HNO_3) and solid copper (wires), producing both nitrogen dioxide (NO_2) and NO. The nitrogen dioxide is then directed into a second reaction flask where it is captured by sodium hydroxide (NaOH). Following this, NO is pumped into the incubation chamber until a concentration of 5 ppm is reached, as indicated by the NO detector. Once the desired concentration is achieved, all connections are tightly sealed, and the system is maintained for 1 hour. Reproduced with permission from [50].

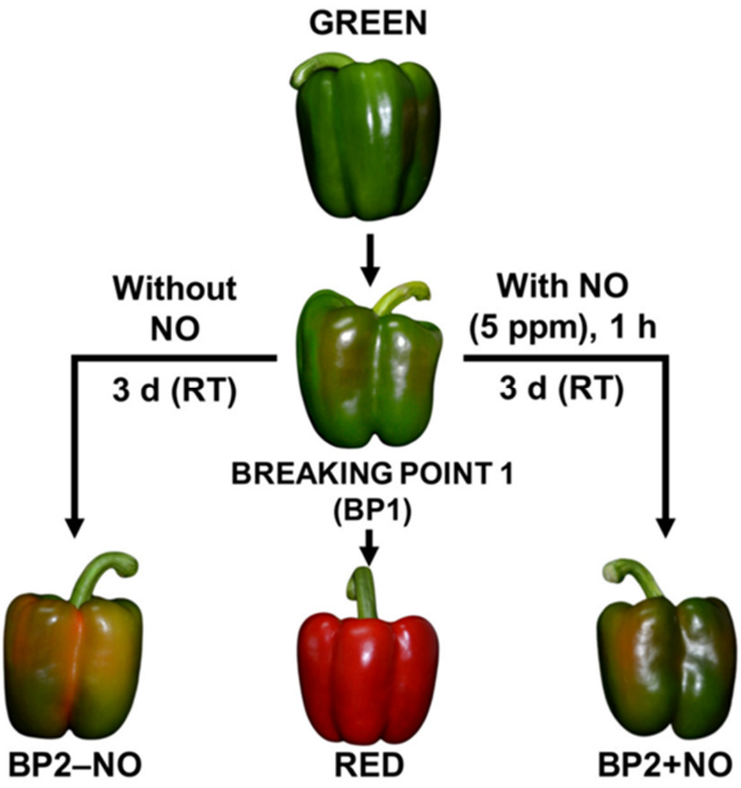

Figure S2. Picture showing the experimental design used in this study with the representative phenotype of sweet pepper (*Capsicum annuum* L.) fruits at different stages and treatments: immature green, breaking point 1 (BP1), breaking point 2 without NO treatment (BP2 – NO), breaking point 2 with NO treatment (BP2 + NO), and ripe red. Fruits were subjected to a NO-enriched atmosphere (5 ppm) in a hermetic box for 1 h and were then stored at room temperature (RT) for 3 days. Reproduced with permission from [39].

Table S1. Identifiers of the different Prxs of different species used to develop the phylogenetic tree of the Prxs proteins.

Plant species	Prx	Protein ID	Reference genome
Arabidopsis thaliana	AtPrx2B<br>AtPrx2F<br>AtPrx2-Cys BAS1<br>AtPrx1<br>AtPrxQ<br>AtPrx1-Cys<br>AtPrx2E<br>AtPrx2-Cys BAS2	at1G65980.1<br>at3G06050.1<br>at3G11630<br>NP_001322441.1<br>at3G26060.1<br>at1G48130<br>at3G52960<br>AAM62760.1	[7]<br>TAIR10.1 (2018)
**Pepper (*Capsicum annumm*)**	CaPrx2B<br>CaPrx2F<br>CaPrx2-Cys BAS1<br>CaPrx1<br>CaPrxQ<br>CaPrx1-Cys<br>CaPrx2E<br>CaPrx2-Cys BAS2	XP_016540190.1<br>XP_016540565.1<br>XP_016546376.1<br>XP_016569570.1<br>XP_016580227.2<br>XP_016548730.1<br>XP_016543463.1<br>XP_016543590.1	UCD10Xv1.1(2018)
**Rice (*Oryza sativa*)**	AtPrx2B<br>OsPrx2F<br>OsPrx2-Cys BAS1<br>OsPrx1<br>OsPrxQ<br>OsPrx1-Cys<br>OsPrx2E<br>OsPrx2-Cys BAS2	AAG40130.1<br>LOC_Os01g16152.1<br>LOC_Os04g33970.1<br>XP_015647991.1<br>LOC_Os06g09610.1<br>LOC_Os07g44430.1<br>BAD53845.1<br>LOC_Os02g33450.2	[41]<br>[95]<br>[9]<br>IRGSP-1.0 (2015)
**Grapevine (*Vitis vinífera*)**	VtPrx2B<br>VtPrx2F<br>VtPrx2-CysBAS1<br>VtPrxQ<br>VtPrx1-Cys<br>VtPrx2E	D7TBK8<br>D7T6T0<br>G1JT83<br>D7TCA6<br>D7T674<br>G1JT87	[96]
**Tomato (*Solanum lycopersicum*)**	SlPrx2B<br>SlPrx2F<br>SlPrx2-CysBAS1<br>SlPrx1<br>SlPrxQ<br>SlPrx2E<br>SlPrx1-Cys<br>SlPrx2-CysBAS2	NP_001234171.1<br>XP_004229422.1<br>NP_001315983.1<br>LOC101266329<br>NP_001296952.1<br>XP_010312248.1<br>XP_004235242.1<br>NP_001315984.1	SL31 (2018)
**Corn (*Zea mays*)**	ZmPrx2B<br>ZmPrxF<br>ZmPrx2-CysBAS1<br>ZmPrx1<br>ZmPrxQ<br>ZmPrx1-Cys<br>ZmPrx2E<br>ZmPrx2-CysBAS2	NP_001232829.1<br>NP_001266601.2<br>NP_001137046.1<br>NP_001353356.1<br>PWZ17293.1<br>PWZ13637.1<br>NP_001148437.2<br>NP_001137046.1	Zm-B73-REFERENCE-NAM-5.0 (2020)
**Potato**	StPrx2B	NP_001275255.1	SolTub_3.0 (2011)

**(*Solanum tuberosum*)**	StPrx2F<br>StPrx2-Cys BAS1<br>StPrx1<br>StPrxQ<br>StPrx1-Cys<br>StPrx2E<br>StPrx2-Cys BAS2	XP_006349225.1<br>XP_006348484.1<br>XP_006356270.1<br>XP_006353774.1<br>XP_006356461.1<br>XP_006339113.1<br>XP_006339159.1	
**Tobacco (*Nicotiana tabacum*)**	NtPrx2B<br>NtPrx2F<br>NtPrx2-Cys BAS1<br>NtPrx1<br>NtPrxQ<br>NtPrx1-Cys<br>NtPrx2E<br>NtPrx2-Cys BAS2	XP_016513674.1<br>XP_016480998.1<br>XP_016471357.1<br>XP_016435750.1<br>XP_016452605.1<br>XP_016505780.1<br>XP_016505666.1<br>NP_001312860.1	Ntab-TN90 (2014)
**Valencian orange (*Citrus sinensis*)**	CsPrx2B<br>CsPrx2F<br>CsPrx2-Cys BAS1<br>CsPrx1<br>CsPrxQ<br>CsPrx1-Cys<br>CsPrx2E<br>CsPrx2-Cys BAS2	XP_006475658.1<br>XP_006489324.1<br>XP_006481573.1<br>XP_006472942.2<br>XP_006474598.1<br>XP_006488766.1<br>XP_006481908.1<br>XP_006481573.1	DVS_A1.0 (2022)
**Poppy (*Papever somniferum*)**	CsPrx2B<br>CsPrx2F<br>CsPrx2-Cys BAS1<br>CsPrx1<br>CsPrxQ<br>CsPrx1-Cys<br>CsPrx2E<br>CsPrx2-Cys BAS2	XP_026417903.1<br>XP_026456514.1<br>XP_026426631.1<br>XP_026404679.1<br>XP_026438752.1<br>XP_026437089.1<br>XP_026427388.1<br>XP_026426631.1	ASM357369v1 (2018)
